# Supplementary material for: Efficacy and safety of manual acupuncture for the treatment of upper limb motor dysfunction after stroke: Protocol for a systematic review and meta-analysis
Source: PLoS One. 2021 Nov 12;16(11):e0258921. doi: 10.1371/journal.pone.0258921 (PMC8589149; doi:10.1371/journal.pone.0258921)
Supplement: Attachment — Submitted filename: Response to Reviewers.doc [file pone.0258921.s002.doc]

**List of Responses**

Dear Editors and Reviewers:

Thank you for your letter and for the reviewers’ comments concerning our manuscript entitled “Efficacy and safety of manual acupuncture in the treatment of Upper Limb Motor dysfunction after stroke: A protocol for systematic review and meta-analysis” (ID: PONE-D-21-03553). Those comments are all valuable and very helpful for revising and improving our paper, as well as the important guiding significance to our research. We have studied comments carefully and have made the correction which we hope meet with approval. The revised portion is marked in red in the paper. The main corrections in the paper and the responses to the reviewer’s comments are as flowing:

Responds to the reviewer’s comments:

Reviewer #1:

1. Response to comment: (……There are some mistakes in English in this document……)

Response: Because English, as a second language, does suggest to you that there are some mistakes in the article. This time, we have carefully revised and checked sentence by sentence, and even applied for professional translation assistance from the translation and editing agency.

Reviewer #2:
1. Response to comment: (Please ascertain that this description of the purpose statement is concise and accurate: “Therefore,...... stroke.”)
Response: ××××××
2. Response to comment: (……The described problem of the lack of high-quality RCTs (please provide some references) is not necessarily solved by conducting a systematic review and meta-analysis. This point should rather be addressed in the discussion section.……)
Response: ××××××

3. Response to comment: (……Some parts (e.g. 2.1.1. Types of participants, 2.8. Sensitivity analysis, etc.)……)
   Response: We have completed the modification according to the suggestions. We mistakenly thought that the repetitive part is that we can quote the previous articles published in BMJopen.
4. Response to comment: (……- Please list the databases used alphabetically……)
   Response:According to the suggestion, we have listed the databases used in this paper in alphabetical order.
5. Response to comment: (……STATISTICAL ANALYSIS
   -How will ……perform statistical tests? Please add detail……)
   Response:We will use Statistics Analysis System and Stata to analyze the Standard Deviation, Standard Error and Mean of each group.

When we encounter the literature with missing data, we will try to contact the author. If we can't get the full data through contact, we will exclude the author's full data.

6.Response to comment: (…… In my personal ……Please provide a rationale or address this topic in the discussion section……)
Response:Because the upper limb motor dysfunction of ischemic stroke occurs in the six stages of Brunnstrom motor function recovery, sometimes electroacupuncture treatment is easy to make the patient's muscle strength lower in the flaccid period and increase abnormally in the spasmodic period. And manual acupuncture can avoid this problem very well. At the same time, it can achieve better curative effect by changing the manual in six different periods.
