# Supplementary material for: Efficacy and safety of manual acupuncture for the treatment of upper limb motor dysfunction after stroke: Protocol for a systematic review and meta-analysis
Source: PLoS One. 2021 Nov 12;16(11):e0258921. doi: 10.1371/journal.pone.0258921 (PMC8589149; doi:10.1371/journal.pone.0258921)
Supplement: Attachment — Submitted filename: Response to Reviewers.doc [file pone.0258921.s003.doc]

**List of Responses**

Dear Prof. Fleckenstein and Reviewers:

Thank you for your letter and for the reviewers’ comments concerning our manuscript entitled “Efficacy and safety of manual acupuncture in the treatment of Upper Limb Motor dysfunction after stroke: A protocol for systematic review and meta-analysis” (PONE-D-21-03553R1). Those comments are all valuable and very helpful for revising and improving our paper, as well as the important guiding significance to our research. We focused on the two main problems that the editor helped us to summarize. First of all, we added a large number of relevant information according to the full text, consulted relevant experts, and completed the discussion part again. Then, we have made great improvements in the language and style of the full text, and invited "editing" organizations to help with native language services.

The revised portion is marked in red in the paper. The main corrections in the paper and the responses to the reviewer’s comments are as flowing:

Responds to the reviewer’s comments:

Reviewer #2:

1. Response to comment: (there are still some remaining in the manuscripts (e.g. high Gao Zhican rate .. the collation of literature, etc.). Please check once again for spelling and language errors.)

Response: First of all, we are sorry that we have not completely checked out spelling and language errors. This time, we have carefully checked spelling and language errors for many times to minimize errors.

2. Response to comment: (Some references are still missing in the manuscript)

Response:Thank you very much for helping us find some references missing. After careful proofreading, we added 9 references in the right place.

We tried our best to improve the manuscript and made some changes in the manuscript. These changes will not influence the content and framework of the paper. And here we did not list the changes but marked in red in revised paper.

We appreciate for Editors and Reviewers’ warm work earnestly and hope that the correction will meet with approval.

Once again, thank you very much for your comments and suggestions.

Authors:Di Cao, Xiaolin Zhang, Mingjun Liu, Qiguang Yang, Shuhong Gu, Tianjiao Gao, Lin Cong, Dehui Ma, Hongju Lin, Shaotao Chen.

E-mail:Shaochenchen3600@163.com.

Institutional: Changchun University of Chinese medicine
