# Supplementary material for: Efficacy and safety of manual acupuncture for the treatment of upper limb motor dysfunction after stroke: Protocol for a systematic review and meta-analysis
Source: PLoS One. 2021 Nov 12;16(11):e0258921. doi: 10.1371/journal.pone.0258921 (PMC8589149; doi:10.1371/journal.pone.0258921)
Supplement: Attachment — Submitted filename: Response to Reviewers.doc [file pone.0258921.s004.doc]

**List of Responses**

Dear Prof. Fleckenstein and Reviewers:

Thank you for your letter and for the reviewers’ comments concerning our manuscript entitled “Efficacy and safety of manual acupuncture in the treatment of Upper Limb Motor dysfunction after stroke: A protocol for systematic review and meta-analysis” (PONE-D-21-03553R1). Those comments are all valuable and very helpful for revising and improving our paper, as well as the important guiding significance to our research.

English is not our mother tongue and may still be not particularly accurate in some expressions, so there are still problems in language expression after the previous three modifications. This time, we specially invited a professional language polishing organization "Yideji" to help with native language services.

The revised portion is marked in red in the paper.

In addition, This is our first time to contribute to our "PLoS One". We found that"Funding Statemen" is different from other magazines. Our actual funding information is as follows:This work was supported by the School construction project of National Administration of traditional Chinese medicine (Grant Number LPGZS22014-11，http://www.satcm.gov.cn/renjiaosi/zhengcewenjian/2018-03-24/1840.html) , The Jilin Local Standard Construction Project (Grant Number: DBXM097-2020，http://scjg.jl.gov.cn/zw/gsgg/202105/t20210526_8082979.html) and the State Administration of Traditional Chinese Medicine of Jilin Province(Grant Number: zybz-zc-2020-004，http://jltcm.jl.gov.cn/tzgg/xgdt/202107/t20210706_8130847.html). Please help to modify it.

We tried our best to improve the manuscript and made some changes in the manuscript. These changes will not influence the content and framework of the paper. And here we did not list the changes but marked in red in revised paper.

We appreciate for Editors and Reviewers’ warm work earnestly and hope that the correction will meet with approval.

Once again, thank you very much for your comments and suggestions.

Authors:Di Cao, Xiaolin Zhang, Mingjun Liu, Qiguang Yang, Shuhong Gu, Tianjiao Gao, Lin Cong, Dehui Ma, Hongju Lin, Shaotao Chen.

E-mail:Shaochenchen3600@163.com.

Institutional: Changchun University of Chinese medicine
